# A Mixed Incoherent Feed-Forward Loop Allows Conditional Regulation of Response Dynamics

**DOI:** 10.1371/journal.pone.0091243

**Published:** 2014-03-12

**Authors:** Szabolcs Semsey

**Affiliations:** Center for Models of Life, Niels Bohr Institute, University of Copenhagen, Copenhagen, Denmark; Tel Aviv University, Israel

## Abstract

Expression of the SodA superoxide dismutase (MnSOD) in Escherichia coli is regulated by superoxide concentration through the SoxRS system and also by Fur (Ferric uptake regulator) through a mixed incoherent feed forward loop (FFL) containing the RyhB small regulatory RNA. In this work I theoretically analyze the function of this feed forward loop as part of the network controlling expression of the two cytoplasmic superoxide dismutases, SodA and SodB. I find that feed forward regulation allows faster response to superoxide stress at low intracellular iron levels compared to iron rich conditions. That is, it can conditionally modulate the response time of a superimposed transcriptional control mechanism.

## Introduction

The highly reactive superoxide (O_2_^-^), which is produced intracellularly as a common byproduct of aerobic life, is one of the main reactive oxygen species that can damage cellular components. Most of the cells exposed to oxygen possess enzymes that provide protection to superoxide toxicity by catalyzing dismutation of superoxide to hydrogen peroxide and oxygen. Extracellular superoxide is produced for instance by phagocytic leukocytes to inactivate invading microorganisms, or by heterotrophic bacteria, affecting global biogeochemistry [1]. Therefore pathogenic bacteria evolved mechanisms to inactivate superoxide molecules or their production in host macrophages [2]. *Escherichia coli* has three superoxide dismutases (SodA, SodB, and SodC) which require different metal cofactors and are regulated in different ways [3]–[8].

The active SodA, or MnSOD, contains manganese. SodA can bind iron and manganese with similar efficiencies [9] but the iron-substituted SodA is catalytically inactive [10]. SodB, or Fe-SOD, contains iron. The cytosolic SodA and SodB proteins have overlapping functions [11] but they are functionally not equivalent [12]. The periplasmic SodC protein contains copper and zinc, and may be important to protect the cell from macrophage killing [13]–[15].

**Figure 1:**
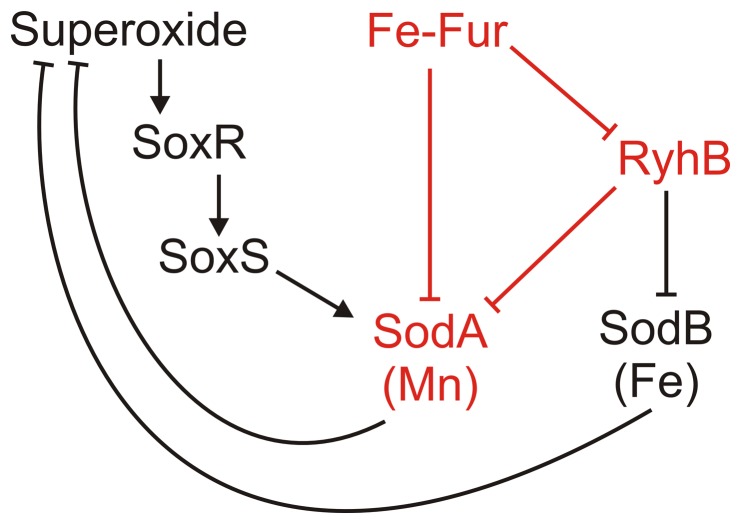
Regulation of cytoplasmic superoxide dismutases by intracellular free iron and superoxide levels. SodA expression is regulated by iron through an incoherent feed-forward loop (shown in red).

Transcription of *sodA*, but not of *sodB*, is activated by superoxide stress through the SoxR-SoxS system [5], [16]. Expression of both SodA and SodB is regulated by intracellular iron availability (Figure 1). Transcription of *sodA* is directly inhibited by the iron-bound form of the Ferric Uptake Regulator (Fe-Fur) [8], while *sodB* transcription is independent of Fe-Fur [7]. However, Fe-Fur levels affect SodB protein production through the small regulatory RNA (sRNA) RyhB, which acts by inhibiting translation initiation and by decreasing mRNA stability [17]. SodA expression is also negatively controlled by RyhB [3], therefore SodA expression is inhibited through an incoherent feed-forward loop (FFL). Similar steady-state SodA activities were found in wild type and Δ*fur* strains [8], suggesting that the FFL results in similar levels of repression at both high and low levels of free intracellular iron (Figure 1). In order to pinpoint the function of this FFL, I investigated the performance of feed-forward regulation of SodA compared to a control system where SodA expression is independent of Fe-Fur and RyhB.

## Results

I have developed a mathematical model to study the characteristics of feed-forward regulation of SodA expression. The model is described in detail in the *Models* section. Using this model I compared the steady state and dynamic behaviors of the natural system, where SodA expression is regulated by RyhB and Fe-Fur through a FFL, with a hypothetical control where SodA production does not depend on FeFur or RyhB concentration.

### The incoherent FFL keeps steady state SodA mRNA levels constant

Similar SodA activities were reported in wild type cells growing in iron rich conditions and in Δ*fur* strains [8] where RyhB is expressed at maximal levels [17]. The parameter values in the model were chosen to match this experimental observation. Thus the model produced similar SodA mRNA levels at high iron (Fe-Fur-repressed) and low iron (RyhB-repressed) conditions.

**Figure 2:**
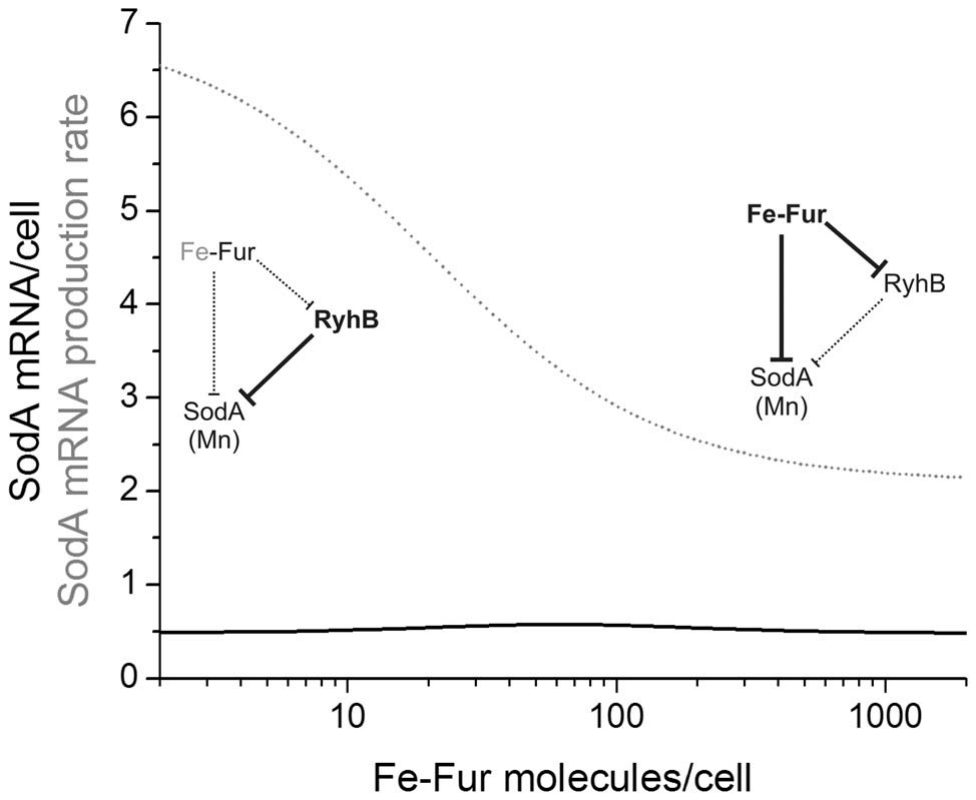
Steady state levels (solid black line) and production rate (dotted grey line) of SodA mRNA as a function of Fe-Fur level in the absence of superoxide stress. The functional interactions in the feed-forward loop at low and high Fe-Fur levels are shown in bold.

First I explored the steady-state levels of SodA at intermediate Fe-Fur levels in the absence of superoxide stress. Simulations showed that SodA mRNA levels were kept in a relatively narrow range as Fe-Fur levels were changed from 2 to 2000 nM (Figure 2). Unlike typical regulatory systems which regulate transitions between a high and a low expression state, i.e. turn a gene on or off, this FFL regulates transitions between similar expression states. Although the SodA mRNA has similar concentrations in these states, it has substantially higher turnover (higher production and degradation rates) at low iron levels (Figure 2, dotted line).

### Simulations of transitions between low and high iron levels

**Figure 3:**
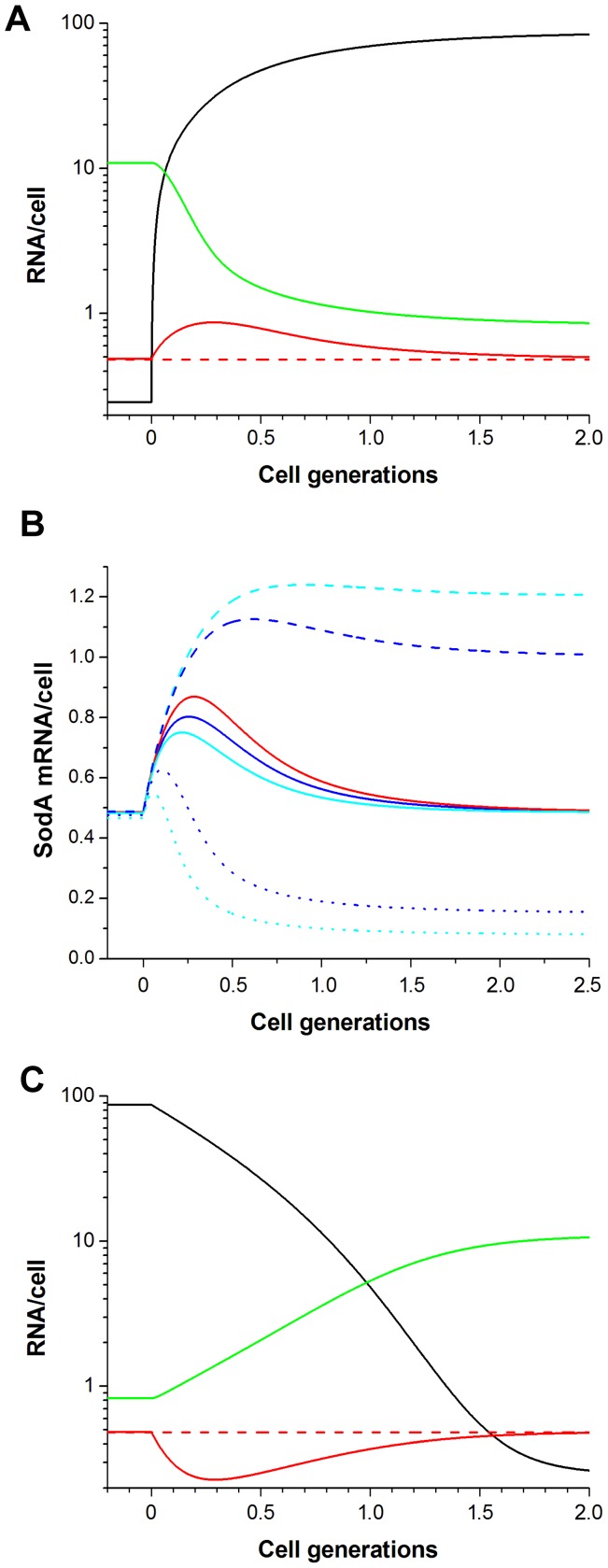
Simulations of transitions from high to low (A and B) and low to high (C) iron conditions in the absence of superoxide stress. The Fe-Fur concentration was changed at zero time from 2000 nM to 1 nM (A and B) or from 1 nM to 2000 nM (C). The solid black curves represent RyhB levels, while the solid red and green curves represent the levels of SodA and SodB mRNAs, respectively. The dashed red line represents the control system where SodA is produced constitutively. (B) Changes in the level of SodA mRNA were simulated using different sRNA production rates (*α_S_*) and RyhB pairing rates (*δ_A_*). The rate pairs used were chosen to obtain the same SodA mRNA levels at low and high iron conditions. The solid red curve represents simulations with the standard parameters (*α_S_* = 280/cell gen; *δ_A_* = 0.0019/min). The solid blue (*α_S_* = 105/cell gen; *δ_A_* = 0.008/min) and cyan (*α_S_* = 70/cell gen; *δ_A_* = 0.016/min) lines show simulations where the sRNA was produced at lower rates but formed a complex with the SodA mRNA at a higher rate. The dashed and dotted lines represent corresponding simulations when only the sRNA production rate was decreased or the complex formation rate was increased, respectively.

To explore how SodA mRNA levels respond to changes in iron availability, I simulated sudden changes in Fe-Fur levels (Figure 3). SodA expression responded by a transient increase to iron depletion (∼2-fold), and returned to its original level after about 1.5 cell generation (Figure 3A). Such transient expression change, i.e. exhibition of near perfect adaptation, is a typical feature of incoherent FFLs [18]–[21]. It results from the delay in RyhB mediated inactivation of SodA mRNA, which depend on the rate of RyhB production relative to its targets production rates and on the efficiency of RyhB pairing with the SodA mRNA. To explore how changes in these parameters affect the shape of the response curve, I performed simulations where RyhB was produced at lower rates than the estimated upper bound used in Figure 3A (see *Methods*). To obtain the same steady-state SodA mRNA levels at low and high Fe-Fur levels as with the standard parameters used in Figure 3A, the rate of RyhB pairing with the SodA mRNA was properly increased (Figure 3B, solid lines). The individual effects of decreased RyhB production rate and increased pairing of RyhB and SodA mRNA were simulated for comparison (Figure 3B, dashed and dotted curves, respectively). As a result of lower RyhB production rate and more efficient complex formation between RyhB and SodA mRNA, the amplitude of the response decreased and the peak of the response occurred earlier. These effects are due to the improved ability of SodA mRNA to compete with the strong targets for RyhB binding [22]. Similar simulations with higher sRNA production rates and decreased pairing rates did not change the response curve.

The opposite effect was observed when the Fe-Fur concentration was suddenly increased (Figure 3C). In this case SodA mRNA levels decreased because Fe-Fur blocks only the production of SodA and RyhB mRNA, and it takes more than one cell generation to clear the existing RyhB sRNA molecules from the system.

### Simulation of superoxide stress at low and high iron levels

**Figure 4:**
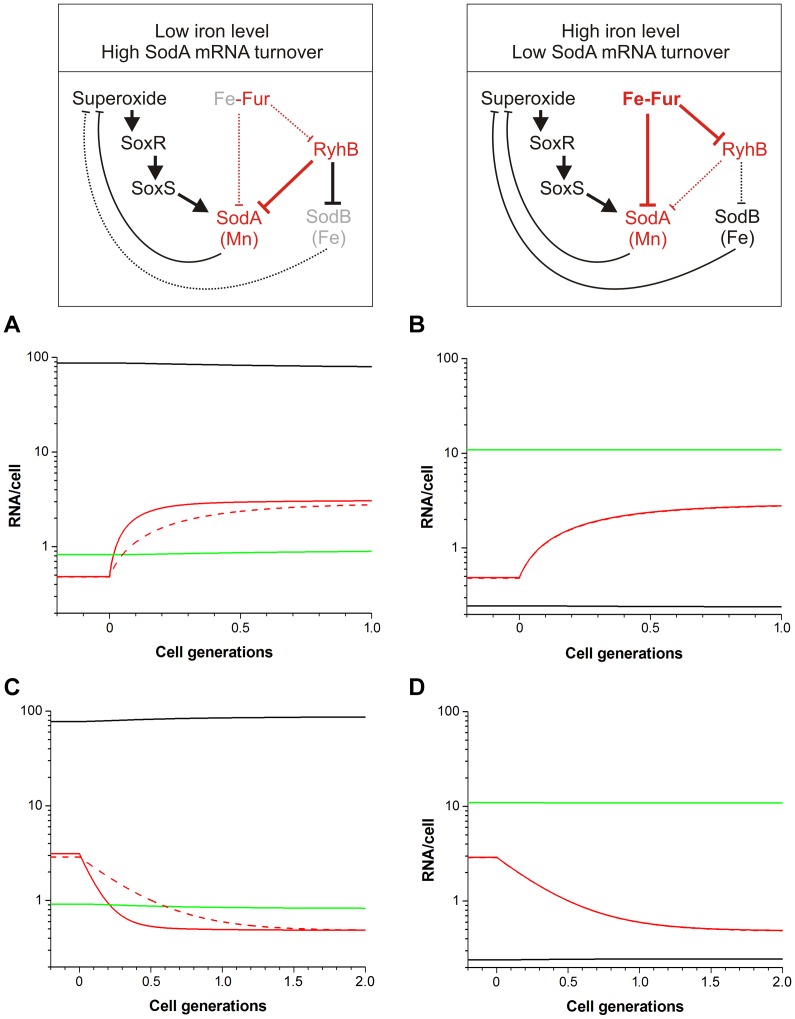
Simulations of changes in superoxide levels at low (left panels) and high (right panels) iron conditions. The dominant regulatory interactions are indicated on the top. At zero time the maximal transcription rate of SodA (*α_A_*) was increased 6-fold (top panels) or decreased from this induced level to the uninduced level (bottom panels). The solid black curves represent RyhB levels, while the solid red and green curves represent the levels of SodA and SodB mRNAs, respectively. The dashed red line, which overlaps with the solid red curves in panels B and C, represents the control system where SodA is produced constitutively.

Intracellular superoxide levels are sensed by the SoxR protein, which contains two [2Fe–2S] clusters. In its oxidized form, SoxR activates transcription of the SoxS protein, which regulates transcription of about 40 promoters. Induction of superoxide stress by paraquat results in about six-fold increase in sodA transcription [5]. Although sodA transcription is increased in Δ*fur* cells, similar fold activation was observed in wild type and Δ*fur* cells in the presence of paraquat [5]. Therefore in the model I assumed that SoxS and Fur act independently on the *sodA* promoter. Because SoxS in intrinsically unstable with an in vitro half-life of about 2 minutes [23], I simulated the effect of superoxide stress simply by increasing the maximal *sodA* transcription rate (Figure 4). I performed simulations at both low (1 nM) and high (2000 nM) Fe-Fur levels. At low Fe-Fur levels the FFL mediated system responded substantially faster than the constitutive system, both to the appearance and to the removal of superoxide stress (Figure 4 A and C). However, in the simulations at high Fe–Fur levels the response dynamics of SodA mRNA was indistinguishable in the FFL mediated and constitutive systems (Figure 4 B and D).

## Discussion

In bacteria, small regulatory RNAs are often part of feed-forward motifs which contain both protein and RNA regulators (mixed FFLs) [24]–[26]. Unlike in pure transcriptional FFL motifs, where incoming regulatory signals must be integrated at the promoter of the target gene using a certain logic [20], [27], [28], in mixed motifs the actions of the protein and sRNA regulators on target production are spatially separated and independent of each other. The function of incoherent feed-forward loops in genetic regulation has been addressed both experimentally and theoretically. For example, incoherent FFLs were shown to accelerate response times [29], provide fold change detection [19], and generate non-monotonic input functions [30].

### Regulatory effects of the mixed FFL

In this work I studied the function of a mixed FFL embedded into a small regulatory network that controls expression of cytoplasmic superoxide dismutases in *E. coli* (Figure 1). This system responds to two intracellular input signals, iron and superoxide concentrations. The system maintains SodA mRNA levels at a narrow concentration range at a wide range of iron concentrations, although the SodA mRNA turnover is higher at low iron levels (Figure 2). Similar to dominant negative autoregulatory systems [31], this FFL is mostly responsible for regulation of expression dynamics during transitions between different environmental conditions.

In the absence of superoxide, the system responds to changes in iron levels with similar pulse dynamics as was previously predicted for incoherent FFLs where the master regulator was an activator [19]. In the iron rich LB medium SodA and SodB transcript levels are similar [32]. However, iron levels greatly exceed manganese levels in *E. coli* grown in iron rich conditions [33], and a fraction of SodA proteins is inactive because being substituted by iron instead of manganese. When intracellular iron becomes scarce, the cell stops production of several non-essential iron using proteins, such as SodB, thus allowing essential proteins to utilize the available limited free iron pool [34].

The loss of SodB activity upon iron depletion is compensated on one hand by a decrease in the level of iron-substituted SodA, and on the other hand by a transiently increased SodA production (Figure 3A). Simulations of the transition from an iron-depleted to an iron rich environment predict the opposite effect (Figure 3C). In this case, SodA activity decreases because of the slower production and of the higher iron substitution rates as well.

### Superimposed global controls

SodA expression is regulated by global regulators responding to intracellular iron and superoxide concentrations. The iron response system is acting through a mixed FFL, involving direct transcriptional and indirect translational regulation, while the superoxide response system acts directly at the transcriptional level (Figure 1). Because the FFL regulating SodA consists of global regulators which are used for regulation of many other genes, it does not generate an extra cost for the cell. Our simulations suggest that the major advantage of this FFL is that it can conditionally modulate the response time of a superimposed transcriptional control mechanism, allowing faster response of SodA when SodB function is limited. At low iron levels the superoxide stress response is predicted to be about three times faster compared to the control system, while at high iron levels the responses are identical (Figure 4). This effect is due to the mixed nature of the FFL, which allows differential regulation of SodA mRNA production and degradation rates by controlling transcription initiation (by Fe-Fur) and translation initiation/mRNA stability (by RyhB) separately. In the FFL regulated system the steady state SodA mRNA level is resulted from equilibrium of production, RyhB mediated degradation, natural degradation, and dilution. Compared to the FFL-regulated system, the constitutive system operates with a constant low SodA mRNA degradation rate, and therefore requires a smaller production rate to obtain the same steady state mRNA level. At low Fe-Fur levels, the FFL regulated system allows faster initial SodA mRNA production rate upon superoxide stress compared to the constitutive system, and reach a steady state quickly because of the increased RyhB mediated degradation, which is directly proportional to the level of SodA mRNA. The FFL also allows faster recovery from stress because of the active degradation of the existing SodA mRNAs.

In conclusion, the FFL allows faster superoxide stress response and better adaptation in iron restricted environments, such as mammalian hosts. The same regulatory system is present in pathogenic strains (e.g. *E. coli* O104:H4, Shigella flexneri 2a str 301), suggesting that the fast response may help in inactivating superoxide molecules generated by the immune system.

## Methods

The dynamical variables I keep track of in our model are the concentrations of RyhB (*S*), SodA mRNA (*m_A_*), and SodB mRNA (*m_B_*). Similar to previous models of sRNA regulation [22], [34]–[37], I assume that: (i) the degradation of the sRNA-mRNA complex is faster than the dissociation of the same complex, so that the binding is effectively irreversible; (ii) both the sRNA and the mRNA are inactivated [38]; (iii) translation of the mRNA is not possible after the complex with the sRNA is formed. I also assume that 1 nM corresponds to one molecule per cell [39], [40].

The deterministic differential equation that model RyhB dynamics is:
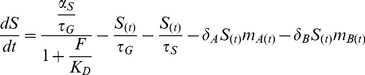
(I)


The first term represents the production of RyhB, which is repressed by Fe-Fur (*F*). In this term, *α_S_* is the maximal production rate of RyhB per cell generation time [41]. *K_D_* ( = 0.02 μM) is the binding constant of Fe-Fur to its operator site [42]. In the second and third terms *τ_S_* ( = 30/ln2 min) [38] and *τ_G_* ( = 40/ln2 min) represents the passive degradation and dilution (by cell devision) of RyhB, respectively. The last two terms represent the active degradation of the RyhB-mRNA complexes by RNaseE [38]. SodA dynamics was modeled by the following equation
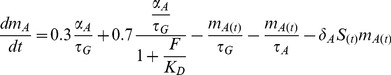
(II)


Based on *sodA* promoter activities reported in wild type and Δ*fur* strains [8], I assume that that the *sodA* promoter has a basal activity even in the presence of Fe-Fur. The first term represents this basal activity, while the second term represents the Fe-Fur regulated activity. The effect of superoxide stress through the SoxR-SoxS system is not modeled explicitly; instead, it is simulated by increasing the maximal *sodA* promoter activity (*α_A_*). The next two terms represent dilution and passive degradation (*τ_A_* = 11.8/ln2 min) [32] of the SodA mRNA, while the last term represents the sRNA mediated degradation. The parameter for RyhB pairing with the SodA mRNA, *δ_A_*, was chosen to be 0.0019/min to obtain similar SodA mRNA levels in the absence and in the presence of Fe-Fur [8].

The dynamics of SodB is modeled by the following equation:
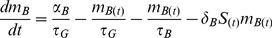
(III)


The first term represents the production of SodB. The next two terms represent dilution and passive degradation (*τ_B_* = 8.8/ln2 min) [32] of the SodB mRNA, while the last term represents the sRNA mediated degradation. The parameter for RyhB pairing with the SodB mRNA, *δ_B_*, was chosen to be 0.014/min to reproduce the experimental observation that the SodB level in *Δfur* cells is 13% of the wild type level [43].

There are several other RyhB target mRNAs exist in the cell, which, similar to SodB, are not regulated directly by Fur. Therefore *m_B_* represents all these mRNAs. The value for *α_B_* (63 molecules per cell generation) was chosen based on the reported level of SodB mRNA (about 5 molecules/cell) [44] and on the estimate that SodB mRNA production constitutes about 40% of the production of strong targets [41].

The values of *α_A_*, and *α_S_* were chosen in such a way that *α_A_*+*α_B_* = *α_S_*/4 (representing the upper bound for *α_S_* [41]), and SodA production was assumed to represent 10% of the production of all targets in the absence of superoxide stress (*α_A_* = 7/cell generation). I compare the above natural system to a hypothetical one where SodA levels depend only on superoxide concentration and not on Fe-Fur or RyhB levels. In this case, equations I and III remain the same, and equation II becomes
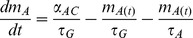
where α_AC_ = α_A_/3.3 to match the Fur-Fe regulated SodA level.
